# Synovial fluid fibrin degradation product can be used as a new auxiliary marker for periprosthetic joint infection diagnosis

**DOI:** 10.3389/fcimb.2025.1435970

**Published:** 2025-05-02

**Authors:** Jincheng Huang, Peng Chen, Zhaodong Zhang, Cheng Cheng, Puji Peng, Yunfei Li, Dongfang Meng, Tao Liu, Yi Jin

**Affiliations:** ^1^ Department of Orthopaedics, Henan Provincial People’s Hospital, Henan University People’s Hospital, Zhengzhou University People’s Hospital, Zhengzhou, Henan, China; ^2^ Department of Orthopaedics, Second Affiliated Hospital of Luohe Medical College, Luohe, Henan, China; ^3^ Department of Orthopaedics, Huaihe Hospital of Henan University, Kaifeng, Henan, China; ^4^ Department of Traditional Chinese Orthopedics, First Affiliated Hospital of Henan University of Traditional Chinese Medicine, Zhengzhou, Henan, China

**Keywords:** prosthetic joint infection, synovial fluid, CRP, ESR, D-dimer, fibrin degradation product, diagnosis

## Abstract

**Background:**

While the value of blood coagulation markers, such as D-Dimer, Fibrinogen, platelet count/mean platelet volume ratio (PC/MPV), and Fibrin Degradation Product (FDP), in the diagnosis of periprosthetic joint infection (PJI) has been explored in recent years, the significance of synovial fluid coagulation markers in PJI diagnosis remains unclear. Therefore, this study aims to investigate the potential value of synovial fluid D-Dimer (sD-Dimer) and synovial fluid FDP (sFDP) in the diagnosis of PJI.

**Materials and methods:**

In a prospective study, the levels of serum C-reactive Protein (CRP), Erythrocyte Sedimentation Rate (ESR), sD-Dimer, and sFDP were measured and compared in 56 patients with PJI (Group A) and 40 patients with aseptic loosening (Group B) who presented at our department from March 1st, 2020, to December 31st, 2023. The diagnostic efficacy of these markers in PJI diagnosis was assessed using the area under the curve (AUC) of the receiver operating characteristic (ROC) curve.

**Results:**

The levels of CRP, ESR, sD-Dimer, and sFDP in Group A were significantly higher than the levels in Group B. The AUC values, optimal threshold values, sensitivity, and specificity for CRP, ESR, sD-Dimer, and sFDP in PJI diagnosis were as follows: CRP [0.920 (95% confidence interval (CI), 0.846–0.965), >6.77, 76.69%, 95.00%], ESR [0.905 (95% CI, 0.828–0.955), >41, 73.21%, 92.50%], sD-Dimer [0.788 (95% CI, 0.692–0.864), >738.65, 66.07%, 80.00%], and sFDP [0.869 (95% CI, 0.785–0.929), >1558.35, 91.07%, 70.00%]. Furthermore, sFDP demonstrated similar performance in PJI diagnosis to CRP and ESR, while sD-Dimer exhibited inferior performance in PJI diagnosis compared to CRP and ESR.

**Conclusions:**

sFDP shows promise as a valuable new adjunctive diagnostic marker for PJI. Further investigations with larger sample sizes are warranted.

## Introduction

Despite the publication of guidelines by the 2010 American Academy of Orthopedic Surgeons (Della Valle et al., 2011), 2013 Musculoskeletal Infection Society (MSIS) (Parvizi and Gehrke, 2014), 2018 International Consensus Meeting (ICM) (Parvizi et al., 2018), 2014 European Bone and Joint Infection Society (EBJIS) (Tande and Patel, 2014), and 2021 EBJIS criteria (McNally et al., 2021) for PJI diagnosis, the timely and accurate diagnosis of periprosthetic joint infection (PJI) remains challenging.

Synovial fluid, also referred to as joint fluid, is situated within the articular joint cavity. It undergoes significant alterations during joint pathology and is considered a crucial component of the diagnostic algorithm for confirming or excluding PJI (Vale et al., 2023). Traditionally, the analysis of synovial fluid has primarily focused on parameters such as synovial leukocyte count, synovial polymorphonuclear percentage (sPMN%), and bacterial culture (Squire et al., 2011). However, the optimal thresholds and diagnostic value of synovial leukocyte count and sPMN% in PJI diagnosis remain subjects of debate (Yi et al., 2015; Pagliaccetti et al., 2021; Abdelaziz et al., 2022). Furthermore, synovial culture has demonstrated only moderate accuracy, with the confirmation or exclusion of PJI often requiring several days of culture (Carli et al., 2019). Consequently, the evaluation of numerous novel synovial markers, including calprotectin (Peng et al., 2022), interleukin-6 (Mihalic et al., 2020), S100 calcium-binding protein A8 (S100A8), S100 calcium-binding protein A9 (S100A9) (Xu et al., 2023), lactate glucose ratio (Berthoud et al., 2020), D-lactate (Karbysheva et al., 2020), and α-Defensin (Zeng et al., 2021) for diagnosing PJI has been undertaken. While some of these emerging synovial markers show promising performance in PJI diagnosis, their widespread adoption in routine clinical practice is hindered by the requirement for specialized antibodies and equipment. Therefore, it is imperative to identify convenient and efficient synovial markers for the diagnosis of PJI.

Although coagulation markers have traditionally been utilized for detecting venous thromboembolism, recent studies have indicated that elevated blood coagulation markers, such as D-Dimer (Shahi et al., 2017; Huang et al., 2019), Fibrinogen (Huang et al., 2021), platelet count/mean platelet volume ratio (PC/MPV) (Paziuk et al., 2020; Sahin et al., 2021) and Fibrin Degradation Product (FDP) (Fujimoto et al., 2018; Xu et al., 2019) may serve as indicators of PJI. Furthermore, research has shown that sFDP and sD-Dimer are markedly expressed in the synovium during inflammatory conditions like rheumatoid arthritis (Anil et al., 2022), and the expression of sD-Dimer is notably elevated in foals with septic joints compared to those without infection (Ewald, 1989). Nevertheless, the applicability of sFDP and sD-Dimer in the diagnosis of PJI remains uncertain.

As C-reactive protein (CRP) and erythrocyte sedimentation rate (ESR) are widely recommended as PJI diagnostic markers in various guidelines (Della Valle et al., 2011; Parvizi and Gehrke, 2014; Tande and Patel, 2014; Parvizi et al., 2018; McNally et al., 2021), the objective of this investigation is to assess the diagnostic utility of sD-Dimer and sFDP in the context of PJI compared with CRP and ESR. Our hypothesis posits that: (i) levels of sD-Dimer and sFDP in PJI patients will exhibit elevation compared to individuals with aseptic loosening; (ii) sD-Dimer and sFDP will demonstrate comparable diagnostic efficacy in PJI detection when compared with CRP and ESR.

## Materials and methods

This study was conducted in strict adherence to the ethical guidelines outlined in the Declaration of Helsinki (Ethical Principles for Medical Research Involving Human Subjects) and received approval from the Ethics Board of Henan Provincial People’s Hospital (Approval No. 202080). Prior to participation, informed consent was obtained from all participants or their legally authorized representatives, ensuring the protection of their rights and welfare throughout the research process.

### Inclusion criteria

The inclusion criteria for this study encompassed patients who presented with either chronic PJI or aseptic loosening and subsequently underwent relevant treatments, including conservative management, debridement, antibiotics, and implant retention (DAIR) surgery, prosthesis removal with antibiotic bone cement spacer implantation surgery, or revision arthroplasty at our institution between March 1, 2020, and December 31, 2023.

### Exclusion criteria

The exclusion criteria for this study comprised patients who met any of the following conditions: 1) a history of anticoagulant therapy within the preceding 2 weeks; 2) recent joint dislocation or trauma occurring within the past 2 weeks; 3) presence of systemic inflammatory conditions, such as rheumatoid arthritis, systemic lupus erythematosus (SLE), psoriasis, polymyalgia rheumatica, or inflammatory bowel disease (IBD); 4) history of hypercoagulable disorders; 5) synovial fluid samples contaminated with blood; 6) initial synovial sample concentrations of sD-Dimer and sFDP falling below the lower limits of detection of the analytical instrument; 7) post-dilution of synovial samples (10-fold, 20-fold, 40-fold and 80-fold dilution were achieved by mixing the sample with respective volume of saline solution.) resulting in sD-Dimer and sFDP concentrations surpassing the upper limits of detection of the instrument; and 8) presence of tumors.

### Study Population

**Figure 1:**
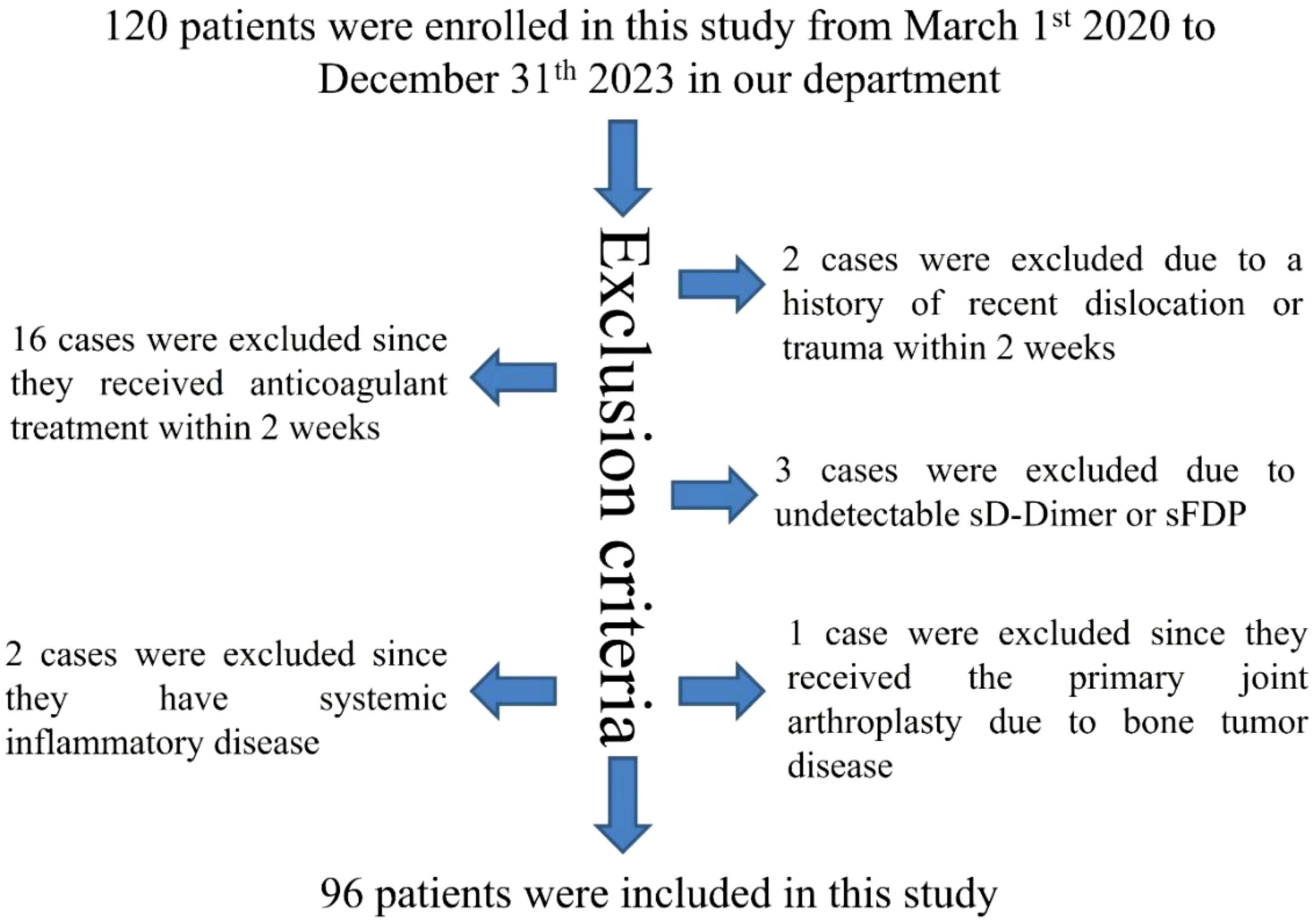
Flow diagram of included patient.

A total of 120 patients were recruited for this study between March 1, 2020, and December 31, 2023, at our department. Among them, 16 cases were excluded due to recent anticoagulant therapy within 2 weeks, 2 cases were excluded for recent joint dislocation or trauma within the same timeframe, 3 cases were excluded for undetectable levels of (sD-Dimer or sFDP, 2 cases were excluded for systemic inflammatory conditions, and 1 case was excluded for undergoing primary joint arthroplasty for bone tumor disease. Ultimately, a cohort of 96 patients met the study’s inclusion and exclusion criteria, as illustrated in **Figure 1**.

### Definition of PJI and aseptic loosening

PJI was defined using the MSIS criteria (Della Valle et al., 2011). Aseptic loosening was defined using the criteria in our previous published paper (Ewald, 1989; Anil et al., 2022).

### Measuring methods

Preoperative levels of serum CRP and ESR were assessed prior to surgery. Synovial fluid samples were obtained either before or during the surgical procedure. Following collection, the synovial fluid samples were placed in tubes containing 3.8% sodium citrate solution (at a ratio of 1:9 citrate to synovial fluid). Within the initial 2 hours post-collection, the samples underwent centrifugation at 1000× g for 10 minutes, after which the supernatants were carefully pipetted and stored at -80°C for subsequent analyses. The concentrations of sD-Dimer and sFDP were determined using an automated coagulation analyzer (Sysmex Europe, CS-5100) with commercially available reagents and controls as per the manufacturer’s instructions. In cases where the concentrations of sD-Dimer or sFDP in the original synovial sample exceeded the upper limits of the analyzer’s detection range, 10-fold, 20-fold, 40-fold and 80-fold dilution were achieved by mixing the sample with respective volume of saline solution. The reported results were adjusted by the corresponding dilution factor.

### Statistical analysis

The normality of quantitative data was initially assessed using the Kolmogorov-Smirnov test. Data conforming to a normal distribution were presented as mean ± standard deviation, and between-group comparisons of quantitative data were conducted using the Independent-sample t test. Non-normally distributed data were expressed as Median (Interquartile Range), and non-parametric methods were employed for between-group quantitative comparisons. Categorical data were compared using the Chi-square test (χ2). All statistical analyses were performed using IBM SPSS Statistics (version 19, IBM SPSS Software).

The diagnostic performance of CRP, ESR, sD-Dimer, and sFDP in PJI diagnosis was evaluated through receiver operating characteristic (ROC) analyses using MedCalc 19.0.4 (MedCalc Software, Ostend, Belgium). Sensitivity, specificity, and the area under the ROC curve (AUC) were key parameters assessed. DeLong’s test (DeLong et al., 1988) was utilized to compare the AUC values between CRP and ESR, CRP and sD-Dimer, CRP and sFDP, ESR and sD-Dimer, as well as ESR and sFDP. A significance level of p < 0.05 was considered indicative of a statistically significant difference.

## Results

### General information of participants

Patients were stratified into two groups: group A (comprising 56 patients with periprosthetic joint infection) and group B (comprising 40 patients with aseptic loosening). Detailed patient demographics are summarized in **Table 1**, showing no significant differences in baseline characteristics between the two groups.

**Table 1:** Comparison of the general data between patients from the two different groups.

Characteristics	Group A (n=56)	Group B (n=40)	P-value
Age (years) ^*^	66.11 ± 8.96	65.00 ± 10.22	0.575
Gender^†^			0.859
Male (n, %)	22 (37.29)	15 (37.50)	
Female (n, %)	34 (60.71)	25 (62.50)	
Joint^†^			0.187
Knee (n, %)	38 (67.86)	32 (80.00)	
Hip (n, %)	18 (32.14)	8 (20.00)	

*The values are given as the mean and standard deviation; ^†^The values are given in terms of number of cases and percentages. P < 0.05 was considered statistically significant.

### Comparison of PJI diagnostic value of CRP, ESR, sD-Dimer and s-FDP

**Figure 2:**
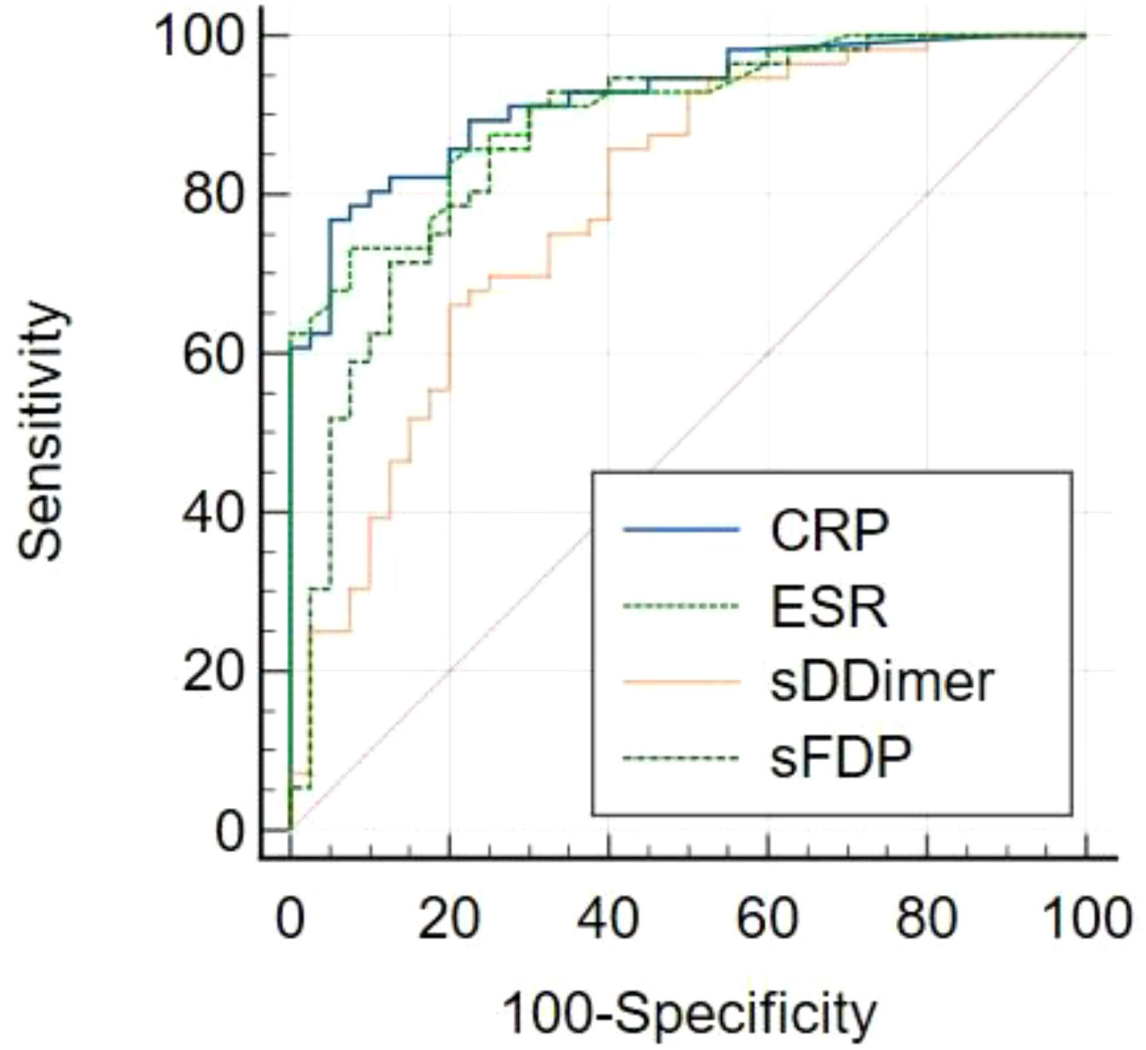
Receiver operating characteristic (ROC) curves of CRP, ESR, sD-Dimer, sFDP in PJI diagnosis.

The levels of CRP, ESR, sD-Dimer, and sFDP in group A were significantly higher than the levels observed in Group B (**Table 2**). The receiver operating characteristic (ROC) curve analysis (**Table 3**) revealed that AUC of both sFDP and sD-Dimer were lower than CRP and ESR in PJI diagnosis. In order to know whether there was significant difference when compared the AUC among sFDP, sD-Dimer, CRP and ESR in PJI diagnosis, DeLong’s test (DeLong et al., 1988) was performed and DeLong’s test indicated that sFDP demonstrated comparable diagnostic performance in PJI diagnosis to CRP and ESR. However, sD-Dimer exhibited inferior diagnostic performance in PJI diagnosis compared to CRP and ESR (**Table 4**; **Figure 2**).

**Table 2:** Comparison of CRP, ESR, sD-Dimer, sFDP between patients from the two different groups.

Characteristics	Group A	Group B	P-value
CRP (mg/L)	Median: 35.18 (IQR: 40.37)	Median: 2.26 (IQR: 1.73)	<0.001^*^
ESR (mm/h)	Median: 66.55 (IQR: 54.00)	18.13 (IQR: 19.50)	<0.001^*^
sD-Dimer (ug/L)	Median: 1044.93 (IQR: 841.35)	454.88 (IQR: 585.99)	<0.001^*^
sFDP (ug/L)	Median: 3621.68 (IQR: 2138.19)	1281.04 (IQR: 1776.26)	<0.001^*^

*The values are given by non-parametric analysis. P < 0.05 was considered statistically significant.

**Table 3:** Diagnostic performance of CRP, ESR, sD-Dimer, sFDP in PJI diagnosis.

	AUC	95% Confidence Interval	Youden index J	Optimal threshold value	Sensitivity (%)	Specificity (%)	P value
CRP	0.920	0.846–0.965	0.7179	>6.77	76.69	95.00	<0.0001
ESR	0.905	0.828–0.955	0.6571	>41	73.21	92.50	<0.0001
sD-Dimer	0.788	0.692–0.864	0.4607	>738.65	66.07	80.00	<0.0001
sFDP	0.869	0.785–0.929	0.6107	>1558.35	91.07	70.00	<0.0001

**Table 4:** Pairwise comparison of ROC curves among CRP, ESR, sD-Dimer, sFDP in PJI diagnosis.

	ESR	sD-Dimer	sFDP
CRP	Z=0.575 P=0. 5651	Z= 2.775 P= 0.0055	Z= 1.152 P= 0.2494
ESR	–	Z= 2.387 P= 0.0170	Z= 0.822 P= 0.4109
sD-Dimer	–	–	Z= 3.297 P=0.0010

## Discussion

The precise diagnosis of PJI in patients lacking typical symptoms, such as sinus tract formation, redness, swelling, fever, or persistent pain, remains a significant challenge for orthopedic surgeons. The search for reliable biomarkers for PJI diagnosis continues to be a focus of research. To our knowledge, this study represents the first investigation into the diagnostic value of sD-Dimer and sFDP in PJI diagnosis. Our findings demonstrate that sFDP performs comparably to CRP and ESR in the diagnosis of PJI. With a calculated optimal threshold value of >1558.35, sFDP exhibits a sensitivity of 91.07% and a specificity of 70.00% in PJI diagnosis. Given the rapid analysis, ease of identification, and cost-effectiveness of FDP detection, our results introduce a novel adjunct marker for PJI diagnosis.

Numerous studies have explored various biomarkers and diagnostic tools for PJI, with a particular focus on synovial fluid markers due to their proximity to the site of infection. For instance, synovial calprotectin has shown high sensitivity and specificity in PJI diagnosis, with some studies reporting sensitivity and specificity values of up to 95% and 97%, respectively (Zhang et al., 2020; Peng et al., 2022; Warren et al., 2022). Karbysheva et al. demonstrated that synovial fluid D-Lactate had a sensitivity of 94.3% and specificity of 78.4% in PJI diagnosis when using Musculoskeletal Infection Society criteria (Karbysheva et al., 2020). Theil et al. showed that synovial fluid pH had a strong correlation with synovial leukocyte count and can potentially serve as a diagnostic marker for chronic PJI (Theil et al., 2022). Chen et al. found that synovial fluid leukocyte esterase can used as a rapid PJI diagnostic tool, with a pooled sensitivity of 87% and specificity of 96% (Chen et al., 2019). Although these studies suggested that synovial fluid biomarkers hold promise as new diagnostic indicators for PJI, most of these published synovial fluid biomarkers often require specialized antibodies and equipment for detection, limiting their widespread adoption in routine clinical practice. Different from most of these published synovial fluid biomarkers, FDP detection is already routinely performed in clinical settings, making sFDP a more accessible and practical option for PJI diagnosis.

It is important to note that while sFDP shows promise as a valuable new adjunctive diagnostic marker for PJI, its relatively modest specificity suggests that it should be used in combination with other biomarkers. The integration of multiple parameters for PJI diagnosis has been explored in other studies, with some reporting improved diagnostic accuracy when combining different biomarkers. Lee et al. found that combining synovial fluid calprotectin with CRP and IL-6 significantly improved PJI diagnostic performance (Lee et al., 2017). Diniz et al. found that combined synovial fluid alpha-2-microglobulin and synovial fluid CRP demonstrated improved PJI diagnostic accuracy (Diniz et al., 2023). Yu et al. found that combined platelet-to-albumin ratio with CRP and ESR had a high PJI diagnostic accuracy, with sensitivity and specificity values of 93.8% and 92.5%, respectively (Yu et al., 2025). All these findings highlighted the potential of combining multiple biomarkers to improve diagnostic accuracy. Therefore, further investigations are needed to explore the synergistic use of sFDP with other established biomarkers.

However, it is imperative to interpret the findings of this study within the context of several limitations. Firstly, our study included a modest cohort of 96 patients, underscoring the need for larger, higher-quality studies to comprehensively assess the diagnostic value of sD-Dimer and sFDP in PJI diagnosis. Secondly, we did not compare the AUC values of sD-Dimer and sFDP with synovial fluid leukocyte count and sPMN% in PJI diagnosis. Consequently, we are unable to definitively ascertain whether the diagnostic performance of sD-Dimer and sFDP in PJI diagnosis surpasses that of synovial fluid leukocyte count and sPMN%. Thirdly, our exclusion criteria encompassed patients with systemic inflammatory diseases, a history of anticoagulant therapy, or recent dislocations or trauma within a two-week period, constituting approximately 20% of patients in our department. This exclusion criterion may somewhat constrain the generalizability of our conclusions in the clinical evaluation of PJI. Fourthly, due to the viscosity of synovial fluid and the relatively elevated concentrations of sD-Dimer and sFDP in PJI patients, there were instances where we had to dilute the synovial fluid with an appropriate volume of saline solution to enhance detection success rates. However, this dilution process may introduce a degree of error in concentration detection. Lastly, since this study primarily focuses on preoperative diagnosis, it did not aim to establish a specific minimum follow-up period. Consequently, it is plausible that some patients who were not operated on in our hospital during the study period may have been diagnosed with PJI at a later stage.

## Conclusion

In conclusion, sFDP emerges as a promising adjunctive parameter for PJI diagnosis. Nonetheless, given its relatively modest specificity, the integration of sFDP with other biomarkers is recommended. The synergistic utilization of multiple parameters warrants exploration in more extensive clinical trials and holds potential utility, especially in cases where diagnoses remain inconclusive. Nevertheless, further comprehensive studies are imperative to validate and refine this combined diagnostic approach.

### Future directions

The findings in this study should be interpreted with caution due to the study’s limitations. Further comprehensive research is essential to confirm these results and refine the diagnostic approach for PJI using synovial fluid markers. The integration of multiple biomarkers, along with larger and more diverse patient populations, will be crucial in enhancing the reliability and clinical applicability of these diagnostic tools.

## Data Availability

The raw data supporting the conclusions of this article will be made available by the authors, without undue reservation.
